# Learning how relationships work: a thematic analysis of young people and relationship professionals’ perspectives on relationships and relationship education

**DOI:** 10.1192/j.eurpsy.2023.801

**Published:** 2023-07-19

**Authors:** S. R. Benham-Clarke, J. Ewing, A. Barlow, T. Newlove-Delgado

**Affiliations:** 1 Public Health and Sports Science; 2 Law School, University of Exeter, Exeter, United Kingdom

## Abstract

**Introduction:**

Relationships in various forms are an important source of meaning in people’s lives that can benefit their health, wellbeing and happiness. Relationship distress is associated with public health problems such as alcohol misuse, obesity, poor mental health, and child poverty, whilst safe, stable, and nurturing relationships are potential protective factors. Despite increased emphasis on relationship education (RE) in schools, little is known about the views of relationship professionals on relationship education specifically, and how this contrasts with the views of young people (YP).

**Objectives:**

This Wellcome Centre for the Cultures and Environments of Health funded Beacon project seeks to fill this gap by exploring their perspectives and inform the future development of relationship education.

**Methods:**

We conducted focus groups with YP (n=4) and interviews with relationship professionals (n=10). The data was then thematically analysed.

**Results:**

Themes from YP focus groups included: ‘Good and bad relationships’; ‘Learning about relationships’; ‘the role of schools’ and ‘Beyond Relationship Education’. Themes from interviews with relationship professionals included: ‘essential qualities of healthy relationships’; ‘how YP learn to relate’ and ‘the role of RE in schools’.

**Conclusions:**

YP and relationship professionals recognised the importance of building YP’s relational capability in schools with a healthy relationship with oneself at its foundation. Relationship professionals emphasised the need for a developmental approach, stressing the need for flexibility, adaptability, commitment and resilience to maintain relationships over the life course. YP often presented dichotomous views, such as relationships being either good or bad relationships, and perceived a link between relationships and mental health. Although not the focus of current curriculum guidance, managing relationship breakdowns and relationship transitions through the life course were viewed as important with an emphasis on building relational skills. This research suggests that schools need improved RE support, including specialist expertise and resources, and guidance on signposting YP to external sources of help. There is also potential for positive relationship behaviours being modelled and integrated throughout curriculums and reflected in a school’s ethos. Future research should explore co-development, evaluation and implementation of RE programmes with a range of stakeholders.

**Disclosure of Interest:**

None Declared

